# Percutaneous cement-augmented short-segment pedicle screw fixation plus percutaneous vertebroplasty for stage III Kummell's disease without neurological symptoms: A case report

**DOI:** 10.1016/j.ijscr.2024.109440

**Published:** 2024-03-05

**Authors:** Xudong Meng, Jiongbiao Zhong, Fan Yang, Jiarui Peng, Jiafu Li, Ye Yuan

**Affiliations:** Orthopedics Department, Yueyang Hospital Affiliated to Hunan Normal University, Yueyang 414000, Hunan Province, China

**Keywords:** Kummell's disease, Percutaneous pedicle screw, Bone cement augmentation, Percutaneous vertebroplasty

## Abstract

**Introduction:**

The incidence of stage III Kummell's disease without neurological symptoms is increasing in elderly patients with osteoporotic thoracolumbar fractures. However, the surgical method is still controversial in this condition. This report presented a case of Kummell's disease in which percutaneous bone cement-augmented short-segment pedicle screw fixation combined with percutaneous vertebroplasty was performed, providing a reference for the surgical approach.

**Case presentation:**

The patient was a 72-year-old female who presented unexplained lower back pain accompanied with limited mobility for the past three months. Based on her medical history, physical examinations, and imaging studies, it was confirmed that she had Kummell's disease in stage III without neurological symptoms. We treated her with percutaneous bone cement-augmented short-segment pedicle screw fixation combined with percutaneous vertebroplasty on the symptomatic vertebrae.

**Clinical discussion:**

The majority of patients with stage III Kummell's disease have severe osteoporosis, which result in failure of the internal fixation and a series of other complications. Maintaining the stability of the internal fixation system is crucial, especially after screwing and subsequent locking. When augmented with bone cement, the grip and pull-out resistance of the percutaneous pedicle screws enhance greatly. Simultaneously, percutaneous vertebroplasty on the symptomatic vertebrae can immediately support the spine unit's stability mechanically and maintain the shape of the vertebrae after reduction.

**Conclusions:**

The percutaneous bone cement-augmented short-segment pedicle screw fixation combined with percutaneous vertebroplasty on the symptomatic vertebrae is an effective treatment for stage III Kummell's disease without neurological symptoms. It can effectively restore the vertebral height, correct the kyphotic deformities, improve spinal canal stenosis, and achieve satisfactory short-term clinical outcomes.

## Highlights

• The majority of patients with stage III Kummell's disease have severe osteoporosis, which result in failure of the internal fixation and a series of other complications.
• Maintaining the stability of the internal fixation system is crucial, especially after screwing and subsequent locking.
• When augmented with bone cement, the grip and pull-out resistance of the percutaneous pedicle screws enhance greatly.
• Simultaneously, percutaneous vertebroplasty on the symptomatic vertebrae can immediately support the spine unit’s stability mechanically and maintain the shape of the vertebrae after reduction.

## 1 Introduction

With the aging population and improvement in diagnosis and treatment, Kummell's disease is becoming more common. Due to vertebral body collapse, there is ischemic necrosis and non-union of the bone within the vertebral body, resulting in persistent pain in the lumbar and back region. The condition can progress to spinal kyphosis and lower limb neurological symptoms, severely affecting the patient's daily life [1,2]. The development of Kummell's disease can be divided into five stages: ① initial injury stage: normal spinal X-ray examination, no clinical symptoms; ② Post-traumatic period: patients may have mild low back pain, but these symptoms do not affect limb function; ③ concealment period: basically asymptomatic, lasting from weeks to months; ④ recurrence period: progressive aggravation of pain in the corresponding area of pathological fracture; ⑤ End stage: persistent kyphosis and spinal cord compression.Conservative treatment for Kummell's disease is generally ineffective, if not timely surgery, eventually lead to kyphosis and spinal stenosis aggravated and even neurological symptoms. Surgical treatment can alleviate pain, promote early mobilization, and improve quality of life [3]. However, there is significant disagreement in the choice of surgical approach for stage III Kummell's disease due to its unique pathological changes and clinical manifestations, especially for cases without neurological symptoms. This study reported the clinical efficacy of percutaneous bone cement-augmented short-segment pedicle screw fixation combined with percutaneous vertebroplasty in the treatment of stage III Kummell's disease without neurological symptoms, providing reference for the selection of treatment methods for this type of Kummell's disease. This case report is reported in line with the SCARE criteria [4].

## 2 Case presentation

### 2.1 Case history

The patient is a 72-year-old retired woman. Her main complaint is unprovoked lower back pain accompanied by limited mobility for three months. The pain can be slightly relieved when lying flat but intensifies during movement or turning over. She has undergone conservative treatments such as pain relief and blood stasis removal, but the effects were not satisfactory. Her lower back pain has worsened over the past two weeks, with no neurological symptoms in both lower limbs, such as pain or numbness. She has a history of hypertension for over ten years, during which she has been taking oral antihypertensive drugs. Her blood pressure has been controlled within the normal range.

### 2.2 Physical examination

Significant tenderness and percussion pain were observed at the paravertebral and intervertebral spaces at the T12 level of the thoracolumbar region, with limited mobility in the thoracolumbar segment. Sensory perception, nerve function, and muscle strength in both lower limbs were found to be normal. Bilateral physiological reflexes were present, and pathological reflexes were negative.

### 2.3 Image analysis

**Fig. 1:**
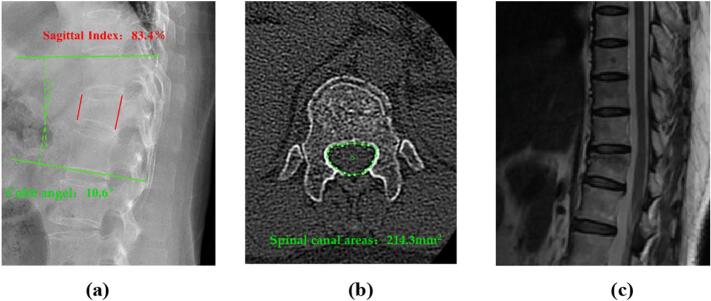
Lateral X-ray, transverse CT and sagittal MRI were performed 3 months before admission.

**Fig. 2:**
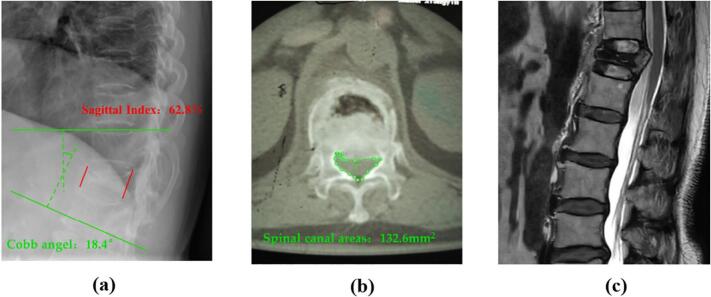
Preoperative lateral X-ray, sagittal MRI and transverse CT were performed.

The patient underwitted X-ray, CT, and MRI examinations three months before admission (Fig. 1). The lateral X-ray image showed a mild compression and posterior convex deformity of the T12 vertebral body, with a Cobb angle of 10.6°and an Sagittal Index of 83.4 %. The transverse CT image showed cortical bone fracture in front of the symptomatic vertebrae, and the horizontal vertebral canal area of the pedicle of 214.3mm^2^. The sagittal T2-weighted MRI image showed compression of the T12 vertebral body and changes in bone marrow edema, without obvious posterior convex deformity or spinal canal stenosis. After admission, follow-up X-ray, CT, and MRI examinations were performed (Fig. 2). The lateral X-ray image showed compression and posterior convex deformity of the T12 vertebral body, with a Cobb angle of 18.4°and an Sagittal Index of 62.8 %. The transverse CT image showed the formation of a fracture line within the symptomatic vertebrae, with posterior wall rupture entering the spinal canal, and the horizontal vertebral canal area of the pedicle of 132.6mm^2^. The sagittal T2-weighted MRI image showed changes in the T12 vertebral body, including compression and a fracture within the vertebral body, along with posterior convex deformity and accompanying spinal canal stenosis.

### 2.4 Operation

The patient was under general anesthesia and in the prone position. The surgical area was sterilized and draped. Using C-arm positioning, the upper and lower vertebrae of the symptomatic vertebrae were located on both sides of the pedicle. A puncture needle (Tianjin Zhengtian) was used for percutaneous pedicle puncture. After satisfactory positioning, the needle core was removed, and a guide wire was inserted. A longitudinal skin incision of about 2 cm was made, and the dilator was expanded step by step. Subsequently, under the guidance of the guide wire, a pedicle screw (Tianjin Zhengtian MIS Plus hollow minimally invasive pedicle screw JJX-IX) was placed. After satisfactory positioning of the screw, under the guidance of C-arm fluoroscopy, a bone cement injector and push rod were used to inject the prepared bone cement (Heraeus OSTEOPAL®V) along the tail end of the pedicle screw, allowing it to diffuse around the screw through the hollow outlet at the front end and the side hole. After the fluoroscopy showed satisfactory diffusion of the bone cement, the bone cement was allowed to solidify. A pre-bent rod of appropriate length was then connected to the screw and tightened with a nut to fix it. The screw was locked in place by the curvature of the connecting rod to reset the diseased vertebra. After resetting the symptomatic vertebrae, percutaneous puncture of the pedicle of the symptomatic vertebrae was performed on one side, with the puncture needle (Shandong Guanlong) finally positioned near the midline of the vertebral body in the anteroposterior view, and in the middle anterior 1/3 in the lateral view. After confirming satisfactory puncture positioning, the bone cement PMMA (Heraeus OSTEOPAL®V) was prepared, and during the wire-drawing period, the bone cement was slowly injected into the vertebral body fissure. The diffusion of the bone cement was observed, and after satisfactory filling, the puncture needle was removed. The tail wing of the screw was broken off, and the incision was cleaned and sutured.

### 2.5 Postoperative management

Patients were advised to rest in bed after surgery, but also to engage in some functional exercises, such as straight leg raises and lower limb flexion and extension exercises. On the third day after surgery, they could carry out corresponding activities under the protection of supports. Antibiotics should be routinely used for infection prevention for 3 days, pain relief and thrombosis prevention for 8 days. Triple therapy for osteoporosis should be administered for 1 year, such as bisphosphonates, calcium agents, and calcitriol.

### 2.6 Follow-up/imaging

**Fig. 3:**
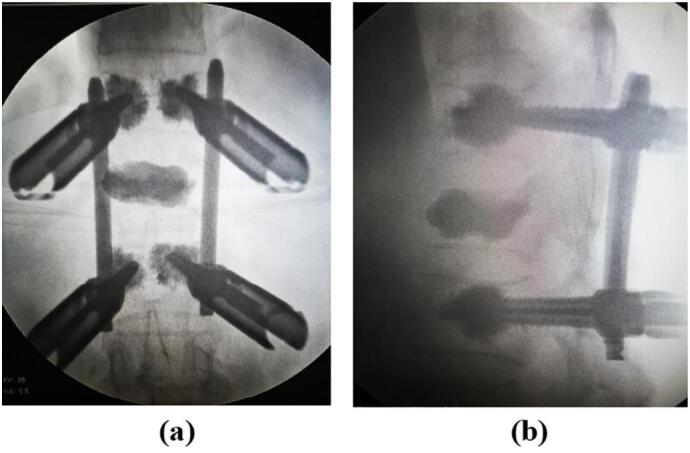
Intraoperative positive and lateral X-ray.

**Fig. 4:**
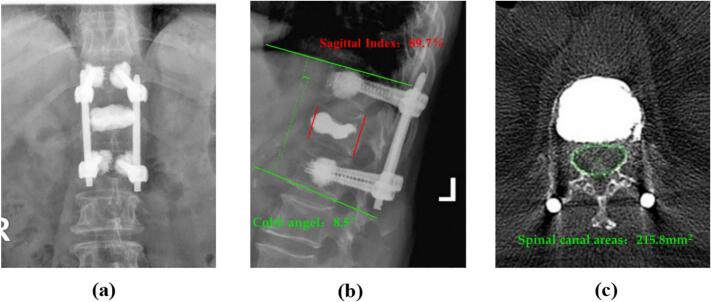
Anteroposterior and lateral X-ray and transverse CT were performed 1 year after operation.

The preoperative visual analogue scale (VAS) score for visual pain simulation was 8.5, and the Oswestry Disability Index (ODI) score was 72.5 %. The VAS score at 1 year postoperative was 2.4, and the ODI score was 25.0 %, indicating significant improvement compared to preoperative scores. Intraoperative X-ray images showed good encapsulation of the screw bone cement, with satisfactory filling and dispersion of bone cement within the T12 vertebral body and no leakage. Correction of the spinal kyphotic deformity was achieved (Fig. 3). The positive and lateral X-ray images at 1 year postoperative demonstrated satisfactory dispersion and filling of the bone cement, with no leakage, and the screw position was satisfactory with no loosening or displacement. The vertebral body height and correction of the kyphotic deformity were satisfactory, with a Cobb angle correction of 8.5°and an Sagittal Index correction of 89.7 %. The transverse CT image at 1 year postoperative showed satisfactory filling of the symptomatic vertebrae body with no leakage, and the horizontal vertebral canal area of the pedicle of 215.8mm^2^, indicating a significant improvement in the degree of stenosis compared to preoperative images (Fig. 4).

## 3 Clinical discussion

This study reported a case of percutaneous bone cement-augmented short-segment pedicle screw fixation combined with percutaneous vertebroplasty for the treatment of stage III Kummell's disease without neurological symptoms, achieving good clinical outcomes. In stage III Kummell's disease, there is severe vertebral body collapse and kyphotic deformity, making percutaneous vertebroplasty ineffective in restoring vertebral height [5]. Additionally, it is prone to cement leakage. By applying pedicle screws to distract and reduce the collapsed vertebral body, not only can the vertebral height be restored, but also the Cobb angle can be corrected, and the spinal canal stenosis in the affected segment can be partially restored. For stage III Kummell's disease with neurological symptoms, decompression surgery is usually combined with long segment fixation and reduction. However, for patients without neurological symptoms, surgeons still prefer to solve the problem through minimally invasive short segment surgery. Nevertheless, the majority of stage III Kummell's disease patients have severe osteoporosis, leading to the failure of internal fixation. Therefore, maintaining the stability of internal fixation has always been a challenging issue faced by spine surgeons.

The trauma of long-segment internal fixation surgery is greater, making it difficult for elderly patients with declining physical condition to tolerate this procedure, and it may also result in the loss of more spinal segment mobility [6]. Increasing the diameter and length of screws, as well as using expansion screws and cement-augmented screws, can all increase the grip strength of the screws [7]. However, increasing the length or diameter of the screws can lead to varying degrees of complications [7]; for patients with severe osteoporosis, methods such as expansion screws and cortical bone channel screws do not achieve ideal stability of the spine [8,9].

In this study, we applied the method of percutaneous pedicle screw fixation augmented with bone cement. On the one hand, the grip strength of the screw was greatly enhanced, increasing its ability to realign the symptomatic vertebrae. The restoration of vertebral morphology, Cobb angle, and spinal canal stenosis were satisfactorily achieved. Meanwhile, the augmentation of the screws with bone cement also improved its resistance to pull-out and stability, thereby maintaining the realigned state of the symptomatic vertebrae. Additionally, bone cement (PMMA) was injected into the symptomatic vertebrae, filling the “void” [10] formed after vertebral realignment and providing immediate support and stability. Combined with the cement-augmented screws, it further maintained the vertebral morphology after realignment and corrected Cobb angle [11], ensuring long-term segmental stability. Therefore, the combination of pedicle screw fixation and percutaneous vertebroplasty for the affected vertebra is an excellent minimally invasive approach. In a study on osteoporotic vertebrae by Liu et al. [12], the pull-out strength of the cement-augmented screws was found to be twice as high compared to non-augmented screws. Wang et al. [13] conducted finite element analysis and biomechanical testing, revealing that the axial pull-out force of the cement-augmented screws was significantly stronger than that of regular screws.

In spinal surgeries involving bone cement, leakage of bone cement may occur due to high injection pressure, premature injection, or excessive injection volume. Patients with stage III Kummell's disease are at high risk for this complication due to severe vertebral compression and co-occurring vertebral posterior wall rupture and collapse. Additionally, these patients often have serious osteoporosis, further increasing their risk. However, the risk of bone cement leakage in this surgical procedure is lower than in PVP, which I believe is due to two reasons: ① During the wire-drawing stage of bone cement injection into the vertebra, the fluidity of the cement is reduced, leading to a more concentrated distribution and effectively preventing leakage [14]; ② After the diseased vertebra is repositioned, the resulting cavity can release pressure caused by vertebral collapse, effectively reducing the risk of bone cement leakage.

## 4 Conclusions

In summary, percutaneous bone cement-augmented short-segment pedicle screw fixation combined with percutaneous vertebroplasty is an effective treatment for stage III Kummell's disease without neurological symptoms. It can effectively restore vertebral height, correct kyphotic deformities, improve spinal canal stenosis, and achieve satisfactory short-term clinical outcomes.

## Consent

Written informed consent was obtained from the patient for publication of this case report and accompanying images. A copy of the written consent is available for review by the Editor-in-Chief of this journal on request.

## Ethical approval

Institution: Yueyang city people's hospital medical ethics committee.

The study is exempt from ethical approval at the research institution.

## Funding

Supported by Hunan Provincial Natural Science Foundation of China (2020JJ9054).

Supported by the scientific research project of Hunan Provincial Health Commission (202104071123).

Supported by the Science and Technology Basic Research Guiding Project of Yueyang City (202012).

## Author contribution

Xudong Meng:Responsible for patient management, postoperative follow-up, data collection and draft writing.

Jiongbiao Zhong:Responsible for disease diagnosis, surgical method selection and implementation, as well as revision of the initial draft.

Fan Yang:Data collection.

Jiarui Peng:Data collection.

Jiafu Li:Data collection.

Ye Yuan:Data collection.

## Guarantor

Jiongbiao Zhong.

## Research registration number

N/A.

## Conflict of interest statement

The authors declare no conflicts of interest.
